# Radiations and female fertility

**DOI:** 10.1186/s12958-018-0432-0

**Published:** 2018-12-16

**Authors:** Roberto Marci, Maddalena Mallozzi, Luisa Di Benedetto, Mauro Schimberni, Stefano Mossa, Ilaria Soave, Stefano Palomba, Donatella Caserta

**Affiliations:** 1 Department of Morphology, Surgery and Experimental Medicine, University of Ferrara, via L. Borsari, 46, 44121 Ferrara, Italy; 2 Faculty of Medicine, University of Geneva, Geneva, Switzerland; 3 Division of Obstetrics and Gynecology, University Hospital of Geneva, Boulevard de la Cluse 30, 1205 Geneva, Switzerland; 4 Department of Medical and Surgical Sciences and Translational Medicine, Sant’Andrea Hospital, Faculty of Medicine and Psychology, University of Rome “Sapienza”, Rome, Italy; 5 Radiation Oncology Unit, S Andrea Hospital, University Sapienza, Rome, Italy; 6 Unit of Obstetrics and Gynecology, Grande Ospedale Metropolitano ‘Bianchi - Melacrino – Morelli’, Reggio Calabria, Italy

**Keywords:** Radiotherapy, Radiation, Infertility, Fertility preservation

## Abstract

Hundreds of thousands of young women are diagnosed with cancer each year, and due to recent advances in screening programs, diagnostic methods and treatment options, survival rates have significantly improved. Radiation therapy plays an important role in cancer treatment and in some cases it constitutes the first therapy proposed to the patient. However, ionizing radiations have a gonadotoxic action with long-term effects that include ovarian insufficiency, pubertal arrest and subsequent infertility. Cranial irradiation may lead to disruption of the hypothalamic-pituitary-gonadal axis, with consequent dysregulation of the normal hormonal secretion. The uterus might be damaged by radiotherapy, as well. In fact, exposure to radiation during childhood leads to altered uterine vascularization, decreased uterine volume and elasticity, myometrial fibrosis and necrosis, endometrial atrophy and insufficiency. As radiations have a relevant impact on reproductive potential, fertility preservation procedures should be carried out before and/or during anticancer treatments. Fertility preservation strategies have been employed for some years now and have recently been diversified thanks to advances in reproductive biology. Aim of this paper is to give an overview of the various effects of radiotherapy on female reproductive function and to describe the current fertility preservation options.

## Introduction

In modern society people are frequently exposed to different types of radiations and this exposure comes form different sources. It could be either related to everyday life (e.g. televisions, mobile phones, computer devices, occupational equipment) or to the necessity of medical care (e.g. diagnostic imaging, interventional radiology procedures, anticancer therapy). Usually radiations are divided into two big subgroups, ionizing and non-ionizing, depending on the energy of the radiated particles.

### Non-ionizing radiations

These type of radiations are basically electromagnetic fields (EMFs) that do not have enough energy to release electrons (non–ionizing), but are able to excite the movement of an electron to a higher energy state. Several classification of EMFs have been proposed, but generally 4 big subgroups are recognized [1, 2]:

(i) extremely low frequency EMFs that have frequencies below 300 Hz (military equipment, railroads)
(ii) intermediate frequency EMFs characterized by frequencies ranging from 300 Hz to 10 MHz (televisions, computer monitors, industrial cables)
(iii) hyper frequency EMFs characterized by frequencies ranging from 10 MHz to 3000 GHz (mobile phones, radio)
(iv) static EMFs that have zero frequency (MRI, geomagnetism)

The biological reaction of the human body to EMFs is still open to discussion, given the fact that many factors can influence the degree to which people may be affected (gender, body mass index, bone density, period of life, frequency and duration of the exposure) [3, 4]. EMFs have a high penetration power that could have disastrous consequences on tissues characterized by high concentrations of ions and electrons. Essentially the effects of non-ionizing radiations can be divided into thermal effects, caused by the heat generated by EMFs on a specific area, and non thermal effects, related to the absorption of the energy of the radiation [5]. Several studies have been published about the cytotoxicity of non-ionizing radiations, but most of them are based on animal models (rats) or on cell line cultures (fibroblasts, melanocytes, lymphocytes, monocytes, muscular cells) [6]. Concerning their effect on the female genital system, different studies on mice have shown that EMFs are able to prevent the formation of antral follicle [7], to inhibit ovulation and to reduce the total number of corpora lutea [8] and given their capacity to extend the lifetime of free radicals they favor cell apoptosis by increasing the oxidative stress resulting in DNA damage [9]. Some authors also observed that mice exposed to non-ionizing radiation have an increased number of macrophages in granulosa cells and lipid drops in theca cells [10]. Combined with the fact that an elevated number of macrophages has also been found in rats’ growing follicles and corpora lutea, some authors postulate that EMFs exposure could accelerate apoptosis in ovarian cortical tissue (responsible for oocytes degeneration) [11]. Some studies conducted on pregnant women have focused on the effect of occupational exposure to video display terminals and pregnancy outcome: an elevated abortion rate and an increased number of birth defects have been found [12, 13]. However, these results need to be carefully considered, because controversial conclusions have been obtained on animal models [14].

### Ionizing radiations

These are high energy radiations that are capable to knock electrons off an electron shell leaving the atoms with a net positive charge (ionization). The biological consequence on human cells of this electron strip is direct or indirect damage of cell’s DNA. In case of a direct damage, the displaced electron breaks the DNA strand, where in case of an indirect damage the electron reacts with a water molecule resulting in the creation of free radicals that in the end damage the cell’s DNA. A double-strand DNA break allows the potential for incorrect DNA repair leading to cell death or for symmetrical translocation, which could lead to the expression of an oncogene during cell replication or to abnormal division in gonads with potential hereditary disorders. The effects of ionizing radiations can be divided into two types:

(i) *deterministic effects,* which are dose-dependent. They take place only once the threshold has been exceeded and cause a functional impairment of a tissue/organ (e.g. impaired fertility related to altered ovarian function);
(ii) *stochastic effects,* which are the result of symmetrical translocation during cell replication. In this case there is no threshold level, but the odds of a stochastic effect to occur increases linearly with the dose (linear no-threshold hypothesis).

Charged particles, X-rays and gamma-rays represent ionizing radiations commonly used in medical care (diagnostic imaging and procedures, radiotherapy).

Given the extent of the topic, this review is focused on the most relevant clinical effects of radiotherapy on female fertility and on possible options for fertility preservation in young cancer survivors.

## Radiation therapy

In Europe, 130,500 new cancer diagnoses (non-melanoma skin cancers being excluded) are made per year in prepubertal children and adolescents. The most common types of cancer occurring in this group of patients include lymphomas (21%), melanomas (15%), cancers of the male genital system (11%), cancers involving the endocrine system (11%) and cancers of the female genital tract (9%) [15]. Nowadays, radiotherapy represents a cornerstone in cancer treatment and, in some cases, constitutes the first therapy proposed both to adolescents and young women (< 45 years old) with different tumors (e.g. sarcomas, medulloblastomas, advanced cervical cancer, rectal cancer, anal cancer and Hodgkin’s lymphomas) [16, 17]. Several types of cancer may require radiation therapy before or after surgery. In women, pelvic irradiation can be recommended in case of cervical cancer, endometrial cancer, bladder cancer and rectal cancer, where cranio-spinal irradiation can be useful in case of central nervous system cancers or haematological malignancies (e.g. Hodgkin’s disease). Total body irradiation is often required before bone marrow transplantation in Hodgkin’s disease [18].

The impact of irradiation on reproductive potential depends on several factors, such as age of the patient, irradiation field, type, dose and duration of the treatment [19]. Pelvic irradiation affects both the ovary and the uterus and cranial irradiation could affect the hypothalamic-pituitary-gonadal axis. Even if reproductive organs’ dysfunctions following radiation may be temporary, the recovery is often unpredictable and in some cases the damage could be permanent [20].

### Ovarian effects

At birth, female ovaries contain approximately 1000,000 non-renewable primordial follicles, the number of which declines over time, primarily through apoptosis and atresia [9]. With age the number of human oocytes peaks at 6–7 million during fetal life (around midgestation) and decreases progressively in quantity and quality and does not regenerate. Approximately 1–2 million oocytes are present at birth, 30,0000–50,0000 at puberty, and 1000 at 51, which is the average age of menopause [21, 22]. Quantity and quality of a woman’s oocytes can be influenced by several factors including genetics, lifestyle, environment, medical procedures and diseases (e.g. endometriosis, ovarian surgery, chemo- and radiotherapy).

During radiotherapy, besides the tumor itself, the radiation field may also include healthy tissues close to the tumor that are unavoidably exposed to radiations. Although in some tissues the damage is reversible, in the ovary it is progressive and permanent. Radiation therapy is commonly applied because of its ability to control cell growth. Generally, cells with high mitotic activity and active DNA replication are more vulnerable to radiation-induced damage, whereas those with low mitotic division rates appear to be more resistant to it. However, oocytes seem to be an exception to this general rule: although arrested at the diplotene stage of the first meiotic division, they are extremely sensitive to radiations. In the past it was thought that the oocyte was not able to repair the genomic damage induced by ionizing radiations because of a lack in DNA repair mechanisms. However, recent studies conducted on animal models, have shown that mammalian oocytes have the enzymatic repair capacity to face and correct DNA modifications and that their radiosensitivity is closely linked to their degree of development [23, 24]. Human oocytes also express different DNA repair genes [25], but their function in the repair of radiation-induced genomic damage is still unclear. Indeed, radiotherapy has a profound impact on ovarian function, characterized by follicular atrophy and reduced follicle stores. This can hasten the natural decline of follicles number that therefore leads to impaired ovarian hormones production, uterine dysfunction due to inadequate estrogen exposure, early menopause and infertility [22, 26, 27]. The extent of the damage that occurs in the ovary depends on several factors such as age of the patient (the younger is the patient at the time of radiation, the greater is the damage), exposure dose, exposure time and eventually associated chemotherapy [28]. In prepubertal age the gonads are extremely vulnerable to radiations [22, 29]; it is estimated that ≤ 2 Gy of radiation would destroy half of immature oocytes [30], while 25–50 Gy would produce infertility in a third of young women and in almost all women over 40 years of age [31–34] (Table 1).

**Table 1:** Radiation doses and risk of gonadal failure (High risk: > 80% sterilized; Mild risk: 20–80% sterilized; Low risk: < 20% sterilized)

Radiation Doses	Risk of Ovarian Failure		
	Prepubertal girls	15–40 years	> 40 years
Pelvic/abdominal irradiation			
< 6Gy	Mild risk	No adverse effects	No adverse effects
15 Gy	High risk	Low risk	Mild risk
25–50 Gy	High risk	Mild risk	High risk
50–80 Gy	High risk	Mild risk	High risk
> 80 Gy	High risk	High risk	High risk
Cranio-spinal irradiation > 25 Gy	Mild risk	Mild risk	Mild risk
Total body irradiation	High risk	High risk	High risk

Nevertheless, if only one side of the ovary is irradiated, ovarian dysfunction would occur in only half of all patients [35]. Furthermore, the risk of ovarian damage induced by gonadal tissue radiation exposure can be augmented when combined with alkylating chemotherapy drugs such as cyclophosphamide [36].

#### Ovarian reserve assessment

Ovarian reserve testing represents a cornerstone in counseling and selection of treatment in all patients who underwent gonadotoxic regimens. The ideal ovarian reserve test should be non-invasive, affordable, reproducible, rapidly interpretable with high specificity and little inter−/intracycle variability. At the present time, there is no perfect test and common methods to assess the ovarian reserve include:

i) dosage of follicle-stimulating hormone (FSH) and estradiol (E2) levels at day 3 of the menstrual cycle
ii) dosage of anti-mullerian hormone (AMH),
iii) transvaginal ultrasound (antral follicle count).

##### Day 3 FSH

In case of impaired fertility, FSH is normally elevated. FSH an indirect measure of the ovarian reserve and is based on the negative feedback of FSH pituitary secretion. It has a higher inter−/intracycle variability and many studies agree that a normal FSH determination does not exclude ovarian dysfunction [37]. AMH measurement and antral follicle count (AFC) evaluation have been shown to have a higher predictive value when compared to day 3 FSH. To enhance its sensitivity it could be combined with E2 levels measurement [38].

##### AMH

AMH is produced by the granulosa cells of early developing follicles and inhibits the transition from the primordial to the primary follicular stage. Its level is relatively independent of circulating gonadotropins concentration, allowing untimed testing. AMH production declines with age and results undetectable after menopause [39, 40]. Among all ovarian tests it is considered the most stable throughout the menstrual cycle [40–43]. However, this issue remain still debated because some intracycle fluctuations have been reported in several studies [44–46]. The main disadvantage of AMH testing is mainly related to the lack of a standardized international assay method that leads to high intra−/inter-assay variability (laboratory differences, sample stability and storage issues) [47]. Recently, in order to overcome this limitation and to improve precision and sensitivity of the test, new automated AMH assay platforms have been developed and are currently used in Europe and Asia [48].

##### Antral follicle count (AFC)

Numerous studies have demonstrated the usefulness of transvaginal ultrasound, particularly at day 3 of the menstrual cycle. It provides two essential parameters that significantly correlate with ovarian reserve and fertility [49]: the measurement of the ovarian volume and the AFC, which defines the number of follicles between 2 and 10 mm in diameter. AFC is easy to carry out and provides immediate results with a good intercycle reliability. However, the precision of the measurement could be compromised by several factors such as differences in the criteria used for the evaluation of antral follicles, differences in ultrasound technology (resolution, 2D vs 3D) and ultrasound scans carried out by multiple sonographers with different degree of training [50, 51].

Nowadays, AMH testing represents the preferred biomarker to asses the ovarian reserve. In patients who underwent radiation therapy, AMH dosage before and after treatment is considered a useful tool in the selection of future fertility treatment. However, it should be taken into account that up to now there is no evidence that AMH pre-treatment levels can predict subsequent fertility and that the effect of different types of malignancies and treatment regimens on AMH level may vary considerably. Future research in this field is needed.

### Uterine effects, pregnancy outcomes and neonatal comorbidities

Besides ovarian failure, also the uterus could be affected by radiotherapy and the consequent radiation-induced damage could be irreversible. In fact, exposure to radiation during childhood leads to altered uterine vascularization, decreased uterine volume and elasticity, myometrial fibrosis and necrosis, endometrial atrophy and insufficiency. Moreover, ulceration and necrosis last several months, and the damaged tissue may be replaced by dense collagen deposition. The cervix gets quite atrophic and loses its elasticity, especially in older patients [52, 53]. In a study published by Larsen et al., the authors report that cytotoxic treatment during childhood does not affect adult uterine size, but, in contrast, uterine irradiation at young age reduces adult uterine volume. The reported results indicate that cancer survivors with spontaneous menstrual cycles have a diminished ovarian reserve. Therefore, reproductive dysfunctions may occur much earlier than anticipated [54]. In adults, an exposure to Total Body Irradiation (TBI) of 12 Gy is associated with significant uterine damage. During childhood, radiation doses of > 25 Gy focused directly to the uterus appear to induce irreversible damage and so far, there is no consensus on the dose of radiation to the uterus, above which a pregnancy would not be sustainable [55]. In 2014 Teh et al. have suggested that patients receiving > 45 Gy during adulthood and > 25 Gy in childhood should be counseled to avoid pregnancies [52]. In fact, radiation could affect embryo implantation and, at the present time, uterine transplantation represent the only possible option for patients with childbearing desire, who underwent uterine radiation. Actually uterine transplantation is not routinely performed for medical and ethical reasons and until now it has been described only one live birth after this procedure [56]. Irradiation may lead to placental disorders (e.g. placenta accreta or placenta percreta), fetal malposition, preterm labor and premature delivery [56, 57]. Although rare, these alterations in uterine architecture can also increase the risk of uterine rupture [58]. Norwitz et al. showed reduced uterine volume and impaired uterine blood flow in a young woman who had a uterine rupture at 17 weeks of gestation. The patient had a remote history of total body irradiation for bone marrow transplantation for childhood leukemia. Although some radiation effects were observed, the severity of injuries was inferior as expected due to low doses of irradiation [59]. Mueller et al. compared the obstetric outcomes among female survivors of childhood and adolescent cancer to those of women without a history of cancer. The children of cancer survivors were more likely to be preterm and to weigh less than 2500 g, while the risk of malformations or death were not increased [60]. Several studies demonstrated an increased risk of adverse pregnancy and neonatal outcomes associated with prior history of abdominal irradiation [22]. In a study published by Chiarelli et al. cancer patients receiving abdominopelvic radiation with or without surgery were more likely to have low birth weight infants and premature low birth weight infants, with higher perinatal mortality rates when compared to patients treated with surgery alone. Additionally, the likelihood of perinatal infant mortality and low birth weight were significantly related to the radiation dose [61]. The results reported by Signorello and colleagues in 2006 are in line with these findings. Authors report that the offspring of patients treated with high-dose radiotherapy have an increased risk of preterm delivery, low birth weight and small-for-gestational-age births when compared to offspring of patients who did not undergo radiotherapy [62]. In addition, in 2009 the British Cancer Survivor study concluded that female survivors of childhood cancer treated with abdominal radiotherapy have a 3-fold increased risk of preterm delivery, a 2-fold increased risk of low birth weight and a small increased risk of miscarriage [63]. Green et al. reviewed the pregnancy outcome among childhood cancer survivors treated with radio- or chemotherapy [64]. One thousand nine hundred fifteen females with 4029 reported pregnancies were considered. Patients, whose ovaries where in the radiation field or close to it or shielded, showed a higher risk of miscarriage, although not statistically significant. Furthermore, the offspring of patients who received pelvic irradiation were more likely to weight < 2500 g at birth. In addition, several studies conducted on female survivors of Wilms tumor (WT) who underwent radiotherapy during childhood, analyzed the effect of low-dose flank irradiation on pregnancy and neonatal outcomes and demonstrated that they are at increased risk of preterm labor, fetal malposition, and premature delivery of low birth weight infants [65–70]. A recent study published in 2010 evaluated the impact of prior radiotherapy for unilateral WT on pregnancy outcomes [71]. Green and colleagues report that women who received flank radiation therapy during childhood are at increased risk of hypertension complicating pregnancy, fetal malposition, premature labor and that the offspring of these women are at risk for low birth weight at delivery(< 2500 g) and premature birth (< 37 weeks of gestation) [64].

### Hypothalamic-pituitary-gonadal axis effects

Brain tumors constitute approximately 17% of all malignant tumors in patients younger than 20 years of age [72] and radiotherapy plays an important role in the curative and palliative treatment of patients with primary and/or metastatic brain tumors [73]. As survival rates of patients with childhood brain tumors have increased to 75%, the side effects of cancer treatment are of particular importance [74]. Radiations induced anterior pituitary hormone deficiency represents the most common irreversible and progressive long-term complication of anticancer treatment and up to 50% of childhood cancer survivors will deal with an endocrinopathy requiring strict follow up to minimize the subsequent effects on growth, bone density, pubertal development and quality of life [75]. The hypothalamic-pituitary-gonadal axis (H-P-G axis) is a hormone system extremely vulnerable to radiotherapy; indeed, both brain surgery and cranial radiation could determine gonadotropin deficiency [76]. The exact mechanism by which radiations influence the H-P-G axis is still poorly understood. A direct injury to H-P cells, rather than reduced hypothalamic blood flow seems to be the major cause of H-P-G axis dysfunction [77]. This hypothesis is supported by the fact that anterior pituitary hormone deficiencies follow a predictable pattern [78] where the growth hormone (GH) axis is the more radiosensitive (GH levels are reduced more than 90% after irradiation), followed by gonadotropin, adrenocorticotropic hormone (ACTH) and thyroid-stimulating hormone (TSH) axis. This is consistent with a direct radiation-induced selective hypothalamic neuronal and pituitary cell damage rather than a universal insult to the H-P axis [79, 80]. Furthermore, the evolution of these hormone deficiencies in time suggests possible delayed effects of radiotherapy on the development of secondary pituitary atrophy after hypothalamic damage [81–83].

The degree of neurotoxicity of radiation depends on total radiation dose, fraction size and duration of the radiation schedule. These variables determine the so called biological effective dose and as it increases, so does the risk of H-P axis dysfunction [81, 83–85]. To minimize the risk, current irradiation regimens call for several fractions (with no more than 2Gy per fraction) spread out several days/weeks (with no more than 5 fractions per week) [86].

It is well known that the secretion of gonadotropin-releasing hormone (GnRH), FSH, luteinizing hormone (LH), estradiol, progesterone, and prolactin follows a pulsatile rhythm which is responsible for the reproductive hormonal environment [87]. Radiation-induced gonadotropin deficiency depends on irradiation dose and tumor location and has a wide spectrum of clinical manifestations, ranging from subclinical (detected only with GnRH testing) to severe forms. Clinically, significant gonadotropin deficiency is usually a late complication with a cumulative incidence of 20–50% on long-term follow-up, regardless of whether radiation was administered in childhood or during adulthood [86]. However, disturbances of the pulsatile rhythm of FSH/LH production can affect fertility and libido and can disrupt the menstrual cycle.

Precocious puberty can occur after radiation doses of < 30 Gy and it may be caused by a disinhibition of cortical influence on the hypothalamus. Studies on rats have shown that low irradiation doses (5–6 Gy) are associated with lower levels of inhibitory transmitter γ-aminobutyric-acid (GABA) and higher expression of GnRH in the pre-optic area [88, 89]. Therefore, radiation-induced early puberty may be the result of a direct damage to the inhibitory GABA system with subsequent premature activation of GnRH neurons. In humans low irradiation doses (18–24 Gy) are associate with precocious puberty only in girls, where higher doses (25–50 Gy) affect both sexes equally [90, 91].

Hyperprolactinemia is another possible consequence of radiation therapy and is manly related to decreased levels of the inhibitory neurotransmitter dopamine. Mild to modest increase in PRL levels are mostly reported in adult females (20 to 50%) after low radiation doses, but also children can be affected (5% of the cases) [92]. Most of the time elevated levels of PRL do not have clinical manifestation, but occasionally may be responsible for amenorrhea and galactorrhea in women and for delayed puberty in children.

Despite optimal medical and surgical management of pituitary tumors, ovulation-induction therapy with gonadotropins is often required in these women [93]. In a recent questionnaire-based study published by Koustenis et al., the authors evaluated fertility characteristics in brain tumor survivors treated with brain irradiation. They found that patients receiving ≥30 Gy, when compare to those who received 18–29 Gy or 0–17 Gy, reported less pregnancies and showed higher rates of permanent amenorrhea and infertility [94]. In a study published in 2002, Green et al. showed that the relative risk of miscarriage is increased in women who received cranial or craniospinal irradiation and in those patients where the ovaries were within or near the radiation field or within 5 cm of the field edge. On the other hand, the risk of miscarriage appears not to be higher when the ovaries are shielded [64]. Furthermore, these data also suggest that spinal irradiation could harm the pregnancy outcome, but further studies are needed to confirm these results.

### Fertility preservation options

In patients diagnosed with cancer the main concern is, obviously, the treatment of the disease. However, given the increased number of young patients that undergo anticancer therapy, long-term side effects, including infertility, should be taken into account [19]. Before anticancer treatment, oncologists should discuss with their patients the consequences of surgery, radio- or chemotherapy, the infertility risk and the fertility preservation options, in order to established an effective, patient-tailored fertility preservation program [55]. Both chemo- and radiotherapy have a relevant impact on reproductive potential, thus fertility preservation procedures should be carried out before and/or during these treatments. Fertility preservation requires a team effort. It should be managed by an oncology centre that has built a close collaboration between oncologists, fertility specialists, psychologists, and primary care physicians to allow early discussion and to offer a full range of options to these patients. Which method of fertility preservation a woman should choose depends on several factors, including the type of disease, the treatment required, the age of the patient, whether she has a long-term partner, and whether treatment can be delayed [95]. Around 70–75% of young cancer survivors are interested in parenthood, but the percentage of patients who undergo fertility preservation techniques before or during cancer treatment is significantly lower [96].

#### Gonadal area shielding

Shielding the gonadal area during total body irradiation is the standard procedure to reduce scatter radiation to reproductive organs [34]. In reproductive-aged women diagnosed with cancers requiring pre- or postoperative pelvic or craniospinal radiation, a safe and effective strategy to prevent premature ovarian failure and to optimize future fertility is Ovarian Transposition (OT) [97–99]. OT is a surgical procedure that transfers the ovaries outside the radiation field by suturing them within the paracolic gutter as high and as lateral as possible. It has been demonstrated that fixation of the ovaries of more than 1.5 cm above the iliac crest is the most important factor to preserve the ovaries [100]. The laparoscopic approach seems to be the preferred one, because it is associated to a more rapid recovery and less postoperative pain and has a success rate in preserving ovarian function of 88.6% [99]. However, the risk involved in the surgical procedure should not be underestimated. The most important complications are vascular injury, infarction of the fallopian tube, and ovarian cyst formation [101].

#### Controlled ovarian stimulation

Since young female patients’ fertility may be impaired after radiotherapy, several other strategies have been proposed. Standard fertility preservation strategies, such as embryo and oocyte cryopreservation, have to be performed before anticancer therapy, but in some cases they may not be feasible (urgency to start cancer therapy, financial limitations, no access to specialized reproductive structures). One approach is to harvest oocytes, fertilize them in vitro, and deep-freeze (cryopreserve) the resulting embryos to be thawed and implanted later. Alternatively, oocytes can be frozen directly, although success rates are lower with this method. Another option is to obtain and cryopreserve directly a sample of ovarian tissue. If none of these is possible, oocytes may be obtained from a donor. If oocytes are to be harvested, there is not an optimal number of oocytes that should be retrieved, but cryopreservation of a large number of oocytes allows the clinician to perform multiple attempts at in vitro fertilization, improving the chances of pregnancy. So that more than one ripe egg can be obtained at a time, the patient must undergo a regimen of controlled ovarian stimulation (COS) to achieve multifollicular growth. There are several stimulation protocols, all based on giving pituitary hormones (e.g. agonist or antagonis of GnRH). To promote follicular development recombinant FSH or human menopausal gonadotropin could be given. A single dose of human chorionic gonadotropin (hCG) is administered to induce ovulation when the lead follicles have reached 18 to 20 mm in size [102]. COS is considered very risky by many oncologists because it takes time and therefore delays anticancer therapy. Moreover, increased levels of circulating estrogens could be harmful in patients with hormone-sensitive cancers [103].

GH is an anabolic hormone with pleiotropic effects and some of its actions are mediated through enhancing serum and local (ovarian) IGF-1 levels and through direct GH receptor (GHR)–mediated effects on the ovary [104–106]. It is well known that GH has many implications in female fertility as many women with GH deficiency suffer a condition of subfertility and require assisted reproductive technologies to conceive. Indeed, poor responder patients with hypogonadotropic hypogonadism co-stimulated with GH showed higher fertilization and pregnancy rates [107–109]. Little is known about the specific molecular pathways implicated in GH effects on the ovary and its possible radioprotective role in ovarian damage induced by γ -irradiation in vivo. It seems that it may activate anti-oxidant systems in order to counteract the oxidative stress-mediated apoptosis [105, 110]. To date, the use of GH before, during or after radiotherapy is not recommended outside of clinical studies.

#### Embryo cryopreservation

Embryo cryopreservation is an established technology that provides a good success rate depending on number and quality of stored embryos [111]. Since a sperm sample is required for oocyte fertilization, the woman should have a partner before starting the treatment [55]. But when a woman is unmarried or does not have a long-time partner, mature oocyte cryopreservation represents another possible option. In fact, oocyte preservation represents a good option for all women who want to maintain their reproductive autonomy [55, 112]. Oocyte cryopreservation can be done either by conventional slow freezing or by vitrification [113, 114]. Vitrification is now the most widely used method due to the improved survival and fertilization rates, compared to the slow freezing method [114, 115]. Neither embryo nor oocyte cryopreservation can be performed in prepubertal girls [55]. Other disadvantages (mostly related to COS) of embryo and oocyte cryopreservation are:

i) risk of thromboembolic phenomena
ii) possible negative effect of COS on estrogen-sensitive tumors
iii) retrieval of a limited number of oocytes/embryos
iv) limited number of future in vitro fertilization (IVF) attempts.

Since in these patients there is generally a limited lapse of time for COS/IVF procedures before starting anticancer therapy, fertility specialists are often tempted to chose heavy stimulation protocols in order to retrieve the maximum number of oocytes. Such a decision should be taken with extreme caution as ovarian hyperstimulation syndrome (OHSS) in these women could be extremely dangerous and could further delay cancer treatment [55, 114, 116]. Indeed, COS for embryo or mature oocyte cryopreservation should be recommended only if the medical condition of the patient allows to carry out the stimulation protocol and the retrieval procedure safely and if there is a fair chance of a good ovarian response. COS takes approximately 2 weeks from the 2nd day of the menstrual cycle, and the implications of delaying cancer therapy to complete the IVF procedure have to be taken into account [55]. Random start of COS has been suggested in order to go beyond this limitation [117]. Furthermore, embryo cryopreservation has also ethical, legal and religious restrictions, especially concerning the disposal of embryos in case of patient death before embryo transfer [116].

### Oocytes cryopreservation

Immature oocytes cryopreservation followed by in vitro maturation is a novel fertility preservation strategy that consists in collecting immature oocytes from primordial follicles (at the germinal vesicle stage) in unstimulated cycles and then letting them mature in vitro. To date, immature oocytes can be cryopreserved before or after in vitro maturation, but several studies have demonstrated that better results are obtained when vitrification follows *the* in vitro maturation process [118]. Compared with mature oocytes, immature oocytes are less susceptible to damage during cryopreservation and thus have a better chance of surviving freezing and thawing, thanks to some peculiar characteristics: they are small, have few organelles, lack a zona pellucida, have low metabolic activity, and are in a state of relative quiescence [119]. Since no ovarian stimulation is required, this technique allows to preserve fertility without delaying the onset of cancer treatment. This option could be considered when cancer treatment cannot be delayed for conventional follicular-phase retrieval [120] or in case of a premature LH surge during ovarian stimulation [121]. However, it is an experimental procedure that should be offered to patients only after proper counseling and as a part of a clinical study [122].

#### Cryopreservation of ovarian tissue

Another possible option to preserve fertility is the cryopreservation of ovarian tissue. It’s a technique that consists in harvesting and freezing ovarian tissue, allowing the preservation of oocytes within primordial follicles [123]. Considering that women at age 30 have about 35 primordial follicles per square millimeter of ovarian tissue, 5 cubic fragments 5 mm wide may be sufficient to obtain more than 4000 primordial follicles [124]. In cases in which complete ovariectomy is necessary, it is possible to remove and cryopreserve fragments of normal ovarian tissue located at the margins of the surgical specimen. After cancer therapy, the ovarian tissue could be transplanted directly into the pelvis in its original location (orthotopic transplant) or outside the pelvis (e.g. fore-arm, subcutaneous abdominal area) (heterotopic transplant). With autotransplantation, there is a high risk of transmission of metastatic cancer cells. Blood-bone cancers such as leukemia and lymphomas are likely to be associated with the highest risk of ovarian metastasis through transplantation of thawed cryopreserved ovarian tissue. Histologic evaluation of ovarian samples before transplantation has been proposed to prevent cancer transmission, although it is not possible to completely abolish this risk [125, 126]. Spontaneous pregnancies can occur after orthotopic pelvic transplant, but IVF is necessary when a heterotopic transplant is carried out [127, 128]. The first successful delivery after an orthotopic autotransplantation of ovarian cortical tissue was reported by Donnez et al. in 2004. The patient had a stage IV Hodgkin’s lymphoma. Biopsy samples of ovarian cortex were taken before anticancer therapy and reimplanted 6 years later. Eleven months after transplantation a viable intrauterine pregnancy was confirmed by an hCG dosage and a transvaginal ultrasound [129]. Another successful delivery after transplantation of cryopreserved ovarian cortical tissue has been recently reported by Rodriguez-Wallberg et al. A 23 years-old woman, diagnosed with Ewing’s sarcoma underwent chemo- (40 weeks intensive high-dose chemotherapy) and radiotherapy (54 Gy pelvic radiotherapy). After the treatment she developed a premature ovarian failure and at the age of 31 years-old she requested a re-transplantation because of childbearing desire. After an orthotopic transplantation the ovarian function promptly recovered and after COS 3 embryos of good quality were obtained. The pregnancy evolved normally and an healthy baby was delivered at term [130]. The first ongoing pregnancy from a heterotopic implantation of ovarian tissue has been reported by Stern et al. in a patient who had both ovaries removed. The tissue was transplanted to the anterior abdominal wall and two oocytes were retrieved after a mild stimulation protocol [131]. No live births have been reported so far in patients who cryopreserved ovarian tissue before puberty [55].

Cryopreservation of ovarian tissue has many advantages over oocyte and embryo cryopreservation:

i) it does not require prior ovarian stimulation with no delay in cancer treatment,
ii) there is no need for a partner or a sperm donor,
iii) it represents the only available option for prepubertal girls diagnosed with cancer,
iv) it preserves a larger pool of follicles and allows spontaneous resumption of the ovarian function [85]. Ovarian function generally resumes between 60 and 240 days post transplant and lasts for up to 7 years [132].

Despite significant advances, to date there have been fewer than 20 babies born worldwide through this method and currently ovarian tissue cryopreservation is still considered experimental and should be recommended only in carefully selected patients [133].

#### Future directions

Recent studies on animal models and in vitro differentiation have suggested mesenchimal stem cells (MSCs) as a possible new option for infertility treatment [134]. MSCs are pluripotent cells, which are able to differentiate into cells of all three germ layer including neuronal cells, osteocytes, chondrocytes, cardiac cells and germ cells [135, 136]. Moreover they have the ability to secrete different growth factors that promote cell survival, proliferation and migration, as well as angiogenesis and immune modulation [137]. MSCs can be collected from different tissues such as bone marrow, umbilical blood, umbilical cord, amniotic fluid, peripheral blood, lung, liver and reproductive tissues [138, 139]. Some studies report the presence on the surface epithelium of the ovaries of very small embryonic like cells (VSELs) that apparently can undergo asymmetric division and generate progenitor germinal cells [140]. The interaction between VSELs and MSCs may play a key role in infertility treatment. In fact, the intercommunication between these two lines of cells may promote the differentiation of MSCs into identical cells of the targeted tissue [141]. In a recent review published in 2018, the authors report that MSCs obtained from bone marrow and umbilical cord are the best candidates for the generation of functional gametes. However, these results should be considered carefully given the difficulty of comparing all the studies published so far (different evaluation methods and different MSCs sources) [142].

In vitro development of primordial follicles is another experimental technique that could further expand the panel of infertility treatment strategies. Previous studies on animal models have shown that primordial murine oocytes developed in vitro can be successfully fertilized and result in viable offspring [143, 144]. In vitro growth and development of human oocytes has also been a matter of research. Telfer et al. demonstrated that activation and growth of human primordial follicles to antral stage is feasible [145] and other groups reported that isolated multi-laminar follicles can resume meiosis and reach Metaphase II [146, 147]. In a recent study published by McLaughlin et al. a new multistep culture system has been proposed in order to achieve complete oocyte development in vitro, from primordial oocytes to metaphase II oocytes [148]. Although a small number of immature oocytes reached Metaphase II, the results obtained in this study make conceivable the use of in vitro generated mature human oocytes for embryo production.

## Conclusion

Modern society cannot avoid human exposure to various types of radiations both in everyday life and occupational activities. The effect of EMFs (non ionizing radiations) on human gametes and, more in general, on female fertility is still poorly understood, but it seems to correlate with higher miscarriage rates and birth defects. On the other hand, many women are exposed to ionizing radiations for medical reasons, both for diagnostic and therapeutic purpose. Radiation therapy can induce long-term side effects on reproductive organs that can impair pubertal development, hormonal regulation, and sexual function, affecting quality of life. Indeed, oocytes are extremely radiosentive and the subsequent depletion of follicle stores caused by radiations exposure may lead to premature ovarian failure, early menopause and infertility. Abdominal irradiation is also associated with alterations in uterine architecture (reduced myomietrial and cervical elasticity with endometrial atrophy/insufficiency) that correlate with several complications during pregnancy (placental disorders, fetal malposition, preterm labor and delivery, low birth weight, higher risk of uterine rupture). Cranio-spinal irradiation is often responsible for late complications on the H-P-G axis. Radiation dose and tumor location influence the wide spectrum of radiation-induced pituitary deficiencies that could range from subclinical manifestations (hyperprolactinemia with no clinical manifestation) to severe forms (precocious puberty, amenorrhea, galactorrhea, elevated abortion rates).

Over the past decade, the great progress in cancer diagnosis and treatment has led to a significant improvement in survival rates, making fertility preservation and quality of life after treatment a key issue, most of all in childhood cancers survivors that should be discussed before starting any radiotherapic treatments. Unfortunately, about 40% of cancer survivors report that they had no fertility counseling at the time of cancer diagnosis. At the present time, a consistent panel of fertility preservation strategies are available (gonadal area shielding, controlled ovarian stimulation, embryos, oocytes and ovarian tissue cryopreservation) and current research, mostly on animal models, is focused on developing further alternatives (mesenchimal stem cells, in vitro maturation of primordial follicles). A multidisciplinary collaboration between oncologists, radiotherapists, gynecologists and fertility specialists is required to improve awareness and availability of these fertility preservation options. Further studies on this field are needed in order to play out a patient-tailored strategy that could restore, when possible, the hormonal function and preserve the reproductive potential of the patient.
